# Perforin Gene Transfer Into Hematopoietic Stem Cells Improves Immune Dysregulation in Murine Models of Perforin Deficiency

**DOI:** 10.1038/mt.2014.242

**Published:** 2015-01-20

**Authors:** Marlene Carmo, Kimberly A Risma, Paritha Arumugam, Swati Tiwari, Adrianne E Hontz, Claudia A Montiel-Equihua, Maria E Alonso-Ferrero, Michael P Blundell, Axel Schambach, Christopher Baum, Punam Malik, Adrian J Thrasher, Michael B Jordan, H Bobby Gaspar

**Affiliations:** 1 Infection, Immunity, Inflammation and Physiological Medicine Programme, Molecular and Cellular Immunology Section, UCL Institute of Child Health, London, UK; 2 Division of Allergy/Immunology, Department of Pediatrics, Cincinnati Children's Hospital Medical Center, University of Cincinnati, Cincinnati, Ohio, USA; 3 Division of Immunobiology, Department of Pediatrics, Cincinnati Children's Hospital Medical Center, University of Cincinnati, Cincinnati, Ohio, USA; 4 Division of Bone Marrow Transplant and Immune Deficiency, Department of Pediatrics, Cincinnati Children's Hospital Medical Center, University of Cincinnati, Cincinnati, Ohio, USA; 5 Institute of Experimental Hematology, Hannover Medical School, Hannover, Germany

## Abstract

Defects in perforin lead to the failure of T and NK cell cytotoxicity, hypercytokinemia, and the immune dysregulatory condition known as familial hemophagocytic lymphohistiocytosis (FHL). The only curative treatment is allogeneic hematopoietic stem cell transplantation which carries substantial risks. We used lentiviral vectors (LV) expressing the human perforin gene, under the transcriptional control of the ubiquitous phosphoglycerate kinase promoter or a lineage-specific perforin promoter, to correct the defect in different murine models. Following LV-mediated gene transfer into progenitor cells from perforin-deficient mice, we observed perforin expression in mature T and NK cells, and there was no evidence of progenitor cell toxicity when transplanted into irradiated recipients. The resulting perforin-reconstituted NK cells showed partial recovery of cytotoxicity, and we observed full recovery of cytotoxicity in polyclonal CD8^+^ T cells. Furthermore, reconstituted T cells with defined antigen specificity displayed normal cytotoxic function against peptide-loaded targets. Reconstituted CD8^+^ lymphoblasts had reduced interferon-γ secretion following stimulation *in vitro*, suggesting restoration of normal immune regulation. Finally, upon viral challenge, mice with >30% engraftment of gene-modified cells exhibited reduction of cytokine hypersecretion and cytopenias. This study demonstrates the potential of hematopoietic stem cell gene therapy as a curative treatment for perforin-deficient FHL.

## Introduction

Hemophagocytic lymphohistiocytosis (HLH) represents a spectrum of inherited and acquired conditions characterized by severe immune dysregulation.^1^ The inherited form is termed familial hemophagocytic lymphohistiocytosis (FHL) and arises from genetic defects that lead to abnormalities of T and NK cell cytotoxicity that in turn result in hypercytokinemia, macrophage activation, hemophagocytosis and a clinical syndrome of fever, coagulopathy, hepatosplenomegaly, and often central nervous system involvement.^2^ Five different genetic loci have now been identified (FHL 1–5), and of these, perforin deficiency (FHL-2) is the most common and best characterized.^3^

In viral infections, effector T and NK cells form an immunological synapse with virally infected target cells. Within the synapse, there is exocytosis of specialized granules that mediate cell cytotoxicity. Perforin is contained within these granules and upon release forms pores in target cell membranes, allowing the passage of granzymes which initiate apoptotic pathways, eventually leading to target cell death.^4^ Mutations in the perforin gene (PRF) lead to failure of pore formation and target cell cytotoxicity in effector cells, resulting in proliferation of highly activated cytotoxic T and NK cells, which in turn lead to hypercytokinemia and the subsequent clinical sequelae of HLH. A murine perforin-deficient model of HLH has been generated which accurately recapitulates the immunological characteristics of the disease.^5^ Correction of immune dysregulation in this model by transplantation with wild-type (WT) bone marrow has been demonstrated, and in these studies, the prevention of HLH development is critically dependent upon the engraftment of functional CD8^+^ T cells.^6^

The prognosis for FHL patients is poor with a median survival of less than 2 months after diagnosis if untreated.^7^ Treatment of FHL-2 is currently limited to the control of infectious triggers and chemotherapeutic regimes to control immune dysregulation, but ~20% of patients do not respond and die as a result of ongoing HLH.^7,8^ The disease can only be corrected by allogeneic hematopoietic stem cell transplantation. Overall survival is best when a matched donor is available and the patient is in clinical remission,^9^ but even then, survival rates vary from 70 to 90% depending on the conditioning regime used.^10,11^ Following mismatched transplants or in patients who are transplanted with active HLH, the outcome following hematopoietic stem cell transplantation is considerably worse with ~50% survival.^7^ The development of alternative strategies based on gene transfer may have significant benefit.

Gene therapy using viral vector–mediated gene transfer into autologous HSCs has been shown to be curative in a number of severe monogenic immunodeficiencies.^12,13,14,15,16^ However, such approaches of “additive” gene transfer have not been attempted for conditions of immune dysregulation, such as HLH, which are substantially different than disorders of immune development, such as severe combined immune deficiency. Based on our results showing that 10–20% WT hematopoietic chimerism is sufficient to reestablish normal immune regulation,^6^ we investigated the feasibility of HSC gene therapy for perforin deficiency. In this report, we show that gene transfer using self-inactivating lentiviral vectors (LVs) results in expression of perforin in T and NK cells and leads to a significant correction of the cytotoxic defects both *in vitro* and *in vivo* in murine models of HLH. Our results suggest that gene therapy may be a promising therapeutic approach for perforin-deficient FHL.

## Results

### LV construction for FLH gene therapy

**Figure 1:**
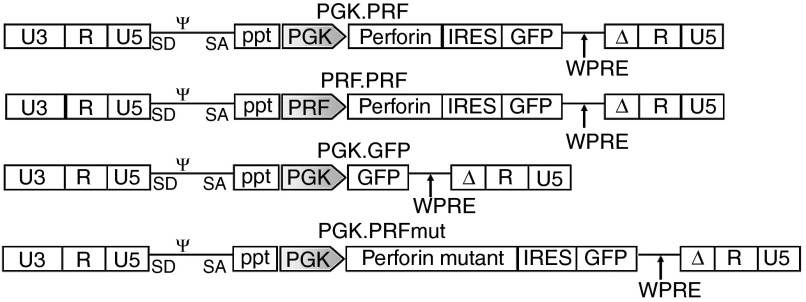
**Schematic representation of self-inactivating perforin lentiviral vectors (LV).** Plasmid configuration is shown. Δ marks SIN deletion with partially deleted U3 of 3′ long terminal repeat. ppt, central polypurine tract; SD/SA, splice donor/splice acceptor; ψ, packaging signal; PGK, phosphoglycerate kinase promoter; PRF, perforin promoter; IRES, internal ribosomal entry site; WPRE, woodchuck hepatitis virus posttranscriptional regulatory element; U3/R/U5, LTR elements.

Two self-inactivating LVs were constructed to promote expression of the human perforin cDNA and GFP under the transcriptional control of either the human phosphoglycerate kinase (PGK) promoter or a lineage-specific human perforin promoter (PRFprom) (PGK.PRF and PRF.PRF; **Figure 1**). A control vector (PGK.GFP) which only expresses GFP and a second control expressing a mutant perforin with null function (PGK.PRFmut) were also generated. The full human PRF promoter is comprised of three regions that span a total of ~5.1 Kb on human chromosome 10 (ref. 17). For this vector, a fragment of this promoter was used consisting of 1.3 Kb DNA upstream of the human perforin gene, which contains the basal core promoter (−244 bp), for expression in effector cells and two elements at −350 and −650 bp that repress transcriptional activity in noneffector cells.^18^ The two functional perforin-expressing vectors (PGK.PRF and PRF.PRF) were tested for expression of GFP and perforin in human cell lines, and high levels of expression were observed in all cell lines using the PGK promoter–driven vector, while expression from the vector with PRFprom was restricted to T (Jurkat) and NK (YT) cell lines (**Supplementary Figure S1**). These results were observed 5 days after transduction and further confirmed 15 days after transduction.

To test for normal perforin expression and processing in a perforin-deficient cell line, we transduced the RBL-1 cell line (rat basophilic leukemia) which is able to process and deliver perforin to secretory granules. Perforin expressed from the PGK.PRF vector exhibited the correct conformation of precursor and mature forms typically noted in lysates from NK and CTL (YT shown for comparison, **Supplementary Figure S2a**). Perforin expression was localized in secretory granules around the cell membrane similar to that seen in YT cells (**Supplementary Figure S2b**).

### Restoration of cytotoxicity *in vitro*

**Figure 2:**
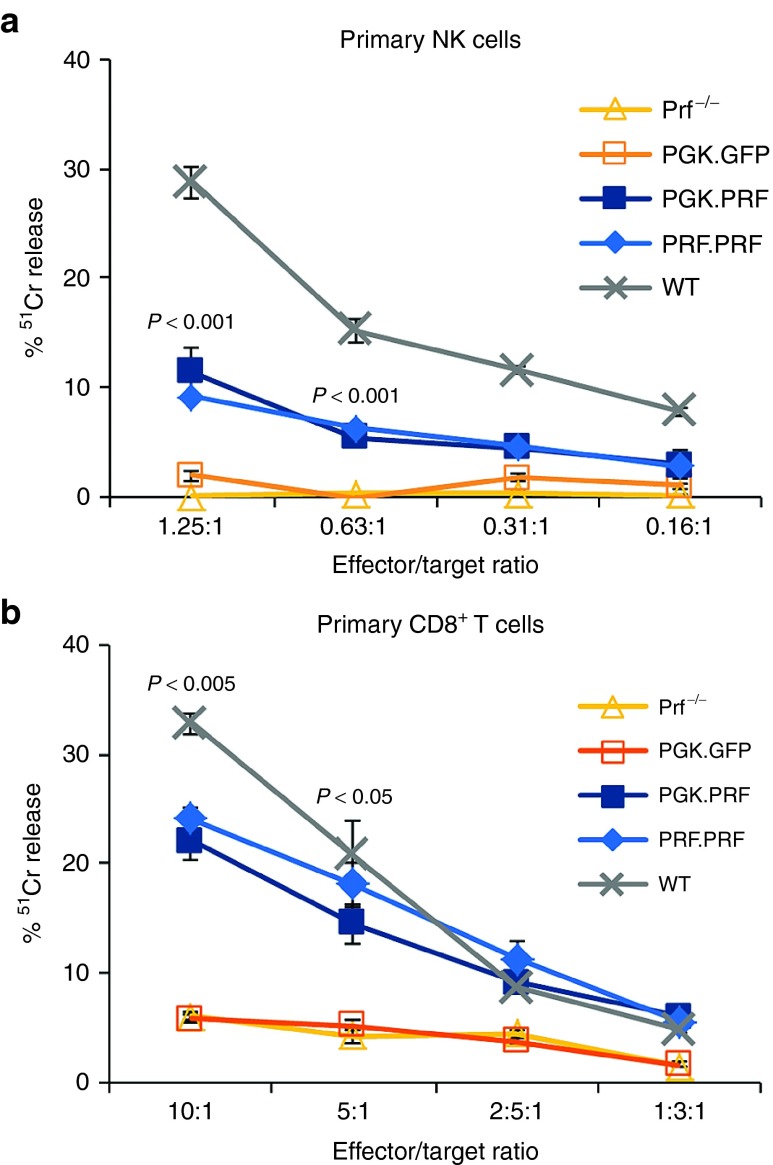
**Perforin reconstitution in peripheral NK and T cells leads to restoration of cytotoxic function.** NK and CD8^+^ T cells from prf^−/−^ mice were transduced with PGK.GFP, PGK.PRF, or with PRF.PRF vectors and processed for chromium assays. (**a**) ^51^Cr release from RMA-S cells co-incubated with transduced and untransduced NK cells and WT mice NK cells. (**b**) ^51^Cr release from anti-CD3 bound P815 cells co-incubated with transduced and untransduced CD8^+^ T cells and WT mice CD8^+^ T cells. The effector to target ratios were normalized taking into consideration the percentage of GFP-positive effector cells (7% in NK cells and 10% in CD8^+^ T cells with both PRF vectors). The *P* values correspond to both the comparisons between the PGK.PRF and the PRF.PRF groups with the prf^−/−^ group. The results presented show one representative experiment from a series of three experiments, and the error bars represent the SEM from the chromium assay triplicates.

To determine if perforin gene transfer could restore cytotoxic function in primary effector cells, splenic NK and T cells derived from perforin-deficient mice (prf^−/−^) were transduced with either the PGK.PRF or PRF.PRF vector. Despite low levels of transduction (GFP expression < 10% with copy numbers of 3 for PGK.GFP, 2.5 for PGK.PRF, and 2 for PRF.PRF), almost full recovery of CD8^+^ T-cell cytotoxicity (redirected killing of P815 cells conjugated with anti-CD3 antibody) was obtained with both vectors when normalized to the number of GFP-positive cells (**Figure 2b**) and recovery of NK cell cytotoxicity to 40–50% of normal was observed (**Figure 2a**). Thus, both vectors were able to restore cytotoxic activity in primary murine perforin-deficient peripheral T and NK cells.

### LV gene transfer into HSCs from perforin-deficient mice

**Figure 3:**
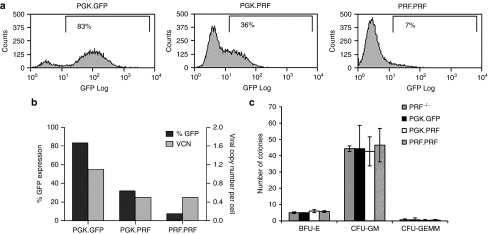
**Efficient lentiviral vector transduction of progenitor cells and demonstration of normal colony formation from transduced cells.** (**a**) Flow cytometry plots showing GFP expression in LSK cells transduced with PGK.GFP, PGK.PRF, or PRF.PRF. (**b**) Transduction efficiency and viral copy number of the LSK cells transduced with PGK.GFP, PGK.PRF, and PRF.PRF. (**c**) The same cells were used in a hematopoietic colony formation assay: BFU-E, burst forming unit erythroid; CFU, colony forming unit granulocyte macrophage; CFU-GEMM, colony forming unit granulocyte, erythroid, macrophage, megakaryocyte. The results presented show one representative experiment from a series of three experiments, and the error bars represent the SEM from the colony formation assay triplicates.

HSC reconstitution of prf^−/−^ mice with lentiviral gene therapy was performed transducing Lin^-^Sca-1^+^c-kit^+^ cells (LSK) with either the PGK.PRF or PRF.PRF or the control PGK.GFP vector. The transduction efficiency obtained using the PGK.PRF, by GFP expression, was 36 and 7% with the PRF.PRF with the same copy number of 0.5 (**Figure 3a**,**b**). This result demonstrates that despite equivalent levels of vector integration, the PRF promoter shows limited expression in progenitor cell lineages (**Figure 3a**,**b**).

### HSC perforin gene transfer does not affect progenitor cell commitment or immune cell development

To assess the risk of toxicity associated with the potential expression or overexpression of perforin in progenitor cells, we investigated the progenitor cell function of transduced LSK cells. The viability of the transduced cells before injecting into mice was above 90% for all three vectors used (PGK.PRF or PRF.PRF or the control PGK.GFP; data not shown). Transduced and untransduced cells were used in hematopoietic colony formation assay (**Figure 3c**), and the number and type of hematopoietic colonies formed was not impaired following transduction by PGK.PRF or PRF.PRF vectors in comparison to untransduced cells or PGK.GFP transduced cells and suggests that perforin expression does not compromise progenitor cell activity.

Three months after injecting mice with gene-corrected cells, the analysis of the blood, thymus, and spleen showed that all mice had normal numbers of NK cells, T cells, B cells, and granulocytes in comparison with WT mice and prf^−/−^ mice (**Supplementary Figure S3**), suggesting that transduction of hematopoietic stem cells with PGK.PRF or PRF.PRF perforin expressing vectors does not inhibit the development of different immune cell lineages.

### HSC gene transfer leads to transgene expression in peripheral cells

**Figure 4:**
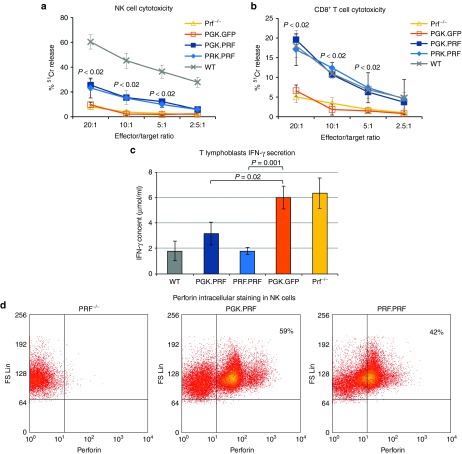
**Lentiviral vector–mediated HSC perforin gene transfer restores T and NK cell cytotoxic function and reduces IFN-γ secretion by T lymphoblasts *in vitro*.** (**a**) ^51^Cr release from RMA-S cells co-incubated with NK cells from mice reconstituted with LSK cells transduced with PGK.GFP, PGK.PRF, and PRF.PRF, prf^−/−^ and WT mice. (**b**) ^51^Cr release from anti-CD3 bound P815 cells co-incubated with CD8^+^ T cells from mice reconstituted with LSK cells transduced with PGK.GFP, PGK.PRF, and PRF.PRF, prf^−/−^ and WT mice. The *P* values correspond to both the comparisons between the PGK.PRF and the PRF.PRF groups with the PGK.GFP group. The highest *P* value is shown for each point. (**c**) IFN-γ production by CD8^+^ lymphoblasts derived from prf^−/−^ mice reconstituted with LSK cells transduced with PGK.GFP, PGK.PRF, and PRF.PRF, from prf^−/−^ and from WT mice after co-incubation with anti-CD3–bound P815 cells for 4 hours. For the three assays *n* = 3 for each group, and the error bars represent the SD. (**d**) Representative flow cytometry dot plots for perforin expression in NK cells from one prf^−/−^ mouse, one mouse reconstituted with PGK.PRF, and one mouse reconstituted with PRF.PRF.

The analysis of GFP expression shows that transduction of LSK cells with PGK.PRF leads to GFP expression in all hematopoietic tissues, between 20% in the bone marrow and 50% in the thymus (**Supplementary Figure S4a**). The expression of GFP in mice reconstituted with the PRF.PRF was higher in CD8^+^ T cells and NK cells, isolated from the spleen and stimulated in culture, confirming preferential expression in effector cells with this promoter (**Supplementary Figure S4b**). For both the PGK.PRF and the PRF.PRF groups, the average copy number in NK cells and CD8+ T cells was 0.4, while in the PGK.GFP group, this copy number was 2.5. Following transduction of LSK cells with either PGK.PRF or PRF.PRF and in contrast to the levels of GFP, the detection of perforin by flow cytometry was low in all cells except NK (**Supplementary Figure S4a,b** and **Figure 4d**). **Figure 4d** shows the perforin staining in NK cells of one representative sample of the PGK.PRF and the PRF.PRF groups. This finding likely reflects the lack of secretory granules required for efficient perforin trafficking and storage in all cells except TCR-stimulated CD8^+^ T cells (CTL) and NK cells.

### HSC perforin gene transfer restores T and NK cell cytotoxic function and inhibits IFN-γ secretion *in vitro*

The major defect caused by perforin deficiency as demonstrated by the prf^−/−^ model is the inability of T and NK cells to kill target cells.^19^ Approximately 3 months following gene modified LSK cell transfer, NK and CD8^+^ T cells were isolated from the spleens of the reconstituted mice and expanded and activated in culture for 5 and 3 days, respectively, prior to testing cytotoxic function by chromium release assays. In both PGK.PRF and PRF.PRF reconstituted mice, the reconstitution of NK cell cytotoxicity was 40% of that seen in WT cells (**Figure 4a**), whereas the recovery of CD8^+^ T cell cytotoxicity (redirected killing of P815 cells conjugated with anti-CD3 antibody) was similar to that seen in WT cells (**Figure 4b**).

Hypercytokinemia is a major feature of the prf^−/−^ model, and the development of HLH disease symptoms is driven by an elevated production of IFN-γ by activated perforin-deficient CD8^+^ T cells *in vivo*.^5^ As a surrogate for *in vivo* challenge, CD8^+^ lymphoblasts cultured from the spleens of the reconstituted mice were used in an IFN-γ assay where the cells were co-incubated with target cells loaded with anti-CD3 antibody. After 4 hours, the supernatant was removed and IFN-γ measured by enzyme linked immunosorbent assay (**Figure 4c**). CD8^+^ lymphoblasts from prf^−/−^ mice exhibit high levels of IFN-γ production. In contrast, perforin-reconstituted CD8^+^ T cells showed significantly reduced IFN-γ, with the cells from mice reconstituted with PRF.PRF transduced cells showing a reduction to levels very similar to that seen in WT cells.

These results demonstrate that the progenitor cell reconstitution of prf^−/−^ mice with perforin expressing LVs leads to a recovery of CD8^+^ lymphoblast function *in vitro*, both in perforin-mediated killing and regulation of IFN-γ secretion. We have observed stable recovery of cytotoxic function in cells from mice which have been engrafted long term (>6 months) with gene-corrected hematopoietic progenitors (data not shown).

### HSC perforin gene transfer corrects antigen-specific CD8^+^ T-cell cytotoxicity in P14 TCR transgenic, prf^−/−^ deficient mice

We next measured antigen-specific CTL function utilizing mice which were perforin deficient and also transgenic for a T-cell receptor specific for the lymphocytic choriomeningitis virus (LCMV)-derived GP33 epitope presented in the context of D^b^ (P14.prf^−/−^) as donor mice for HSC transplantation. HSC from P14.prf^−/−^ mice were transduced with PGK.PRF, or a mutated PRF protein (PRFmut) under the control of the PGK promoter (PGK.PRFmut). PRFmut has a missense mutation (PRF-T435M) that causes a postsynaptic defect in lipid binding allowing protein secretion but not binding of target cells.^20^ LSK transduction efficiencies were equivalent with both vectors (55 and 45% for the PGK vectors expressing WT human PRF or PRFmut, as assessed by GFP expression, data not shown, with copy numbers of 7 and 13, respectively).

**Figure 5:**
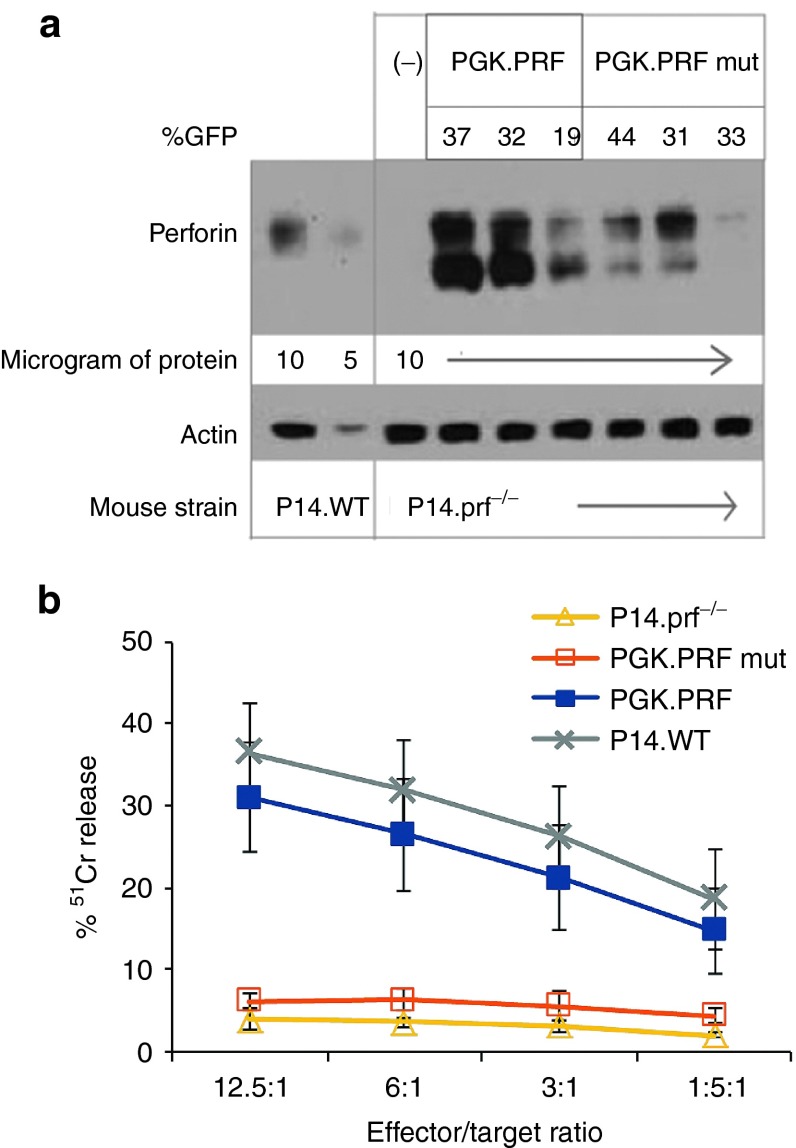
**Lentiviral vector–mediated HSC perforin gene transfer restores PRF expression and corrects antigen-specific CTL killing in prf**^−/−^ **mice.** LV vectors encoding either wild-type human PRF or a mutant human PRF (PRFmut) under the PGK promoter were used to transduce LSK cells from P14.prf^−/−^ mice, which were transplanted into irradiated prf^−/−^ recipients. (**a**) CTL lines derived from these mice were analyzed for GFP expression by flow cytometry, and data is listed as %GFP above the western blot. Perforin expression was analyzed by western blot using Δ G9 anti-perforin Ab. Samples from gene-transduced P14.prf^−/−^ mice were compared to P14.WT and P14.prf^−/−^ mice. (**b**) ^51^Cr release from GP33-loaded EL4 target cells co-incubated with CTL from mice reconstituted with PGK.PRF (*n* = 3) or with PGK.PRFmut (*n* = 3), P14.WT (*n* = 5), and P14.prf^−/−^ mice (*n* = 3).

After stimulation with gp33 peptide for 48 hours and expansion of splenocytes in IL-2 for 3–4 days, cytotoxicity was evaluated against gp33-loaded EL4 target cells. GFP expression in CTL was similar in both WT human PRF and PRFmut expressing mice (ranging from 19 to 44%), and perforin expression was detectable in the CTL of both cohorts (**Figure 5a**). Overall, we see less protein in lines expressing PRFmut because of misfolding.^20^ Antigen-specific cytolysis of targets was detected from gene corrected, CTL expressing WT PRF but not CTL expressing PRFmut (**Figure 5b**). In these gene-corrected CTL, function was equivalent to CTL generated similarly from nontransplanted, perforin sufficient, P14 transgenic mice (P14.WT). Taken together, these data show that gene transfer of a PRF gene into prf^−/−^ HSC can restore cytotoxicity in CTLs recognizing a specific viral antigen.

### HSC perforin gene transfer partially corrects disease phenotype of LCMV-infected prf^−/−^ mice

**Figure 6:**
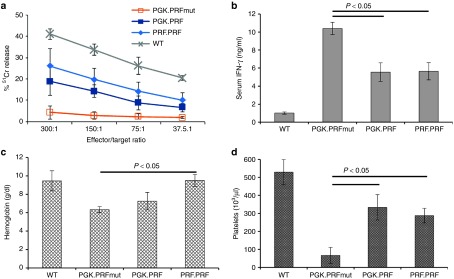
**Perforin gene correction partially restores antigen-specific CTL killing and rescues prf**^**−/−**^ **mice from the development of HLH-like disease after LCMV infection.** Prf^−/−^ mice were irradiated and reconstituted with WT marrow or prf^−/−^ LSK transduced with PGK.PRF, PGK.PRFmut, or PRF.PRF. After 16 weeks, animals were challenged with LCMV infection. (**a**) On day 8 following infection, splenocytes were tested for cytotoxic function by ^51^Cr release assay using GP33-loaded EL4 target cells. Error bars represent SD from four mice/group. (**b**) Day 8 serum IFN-γ levels from animals with ≥30% chimerism of gene corrected (or WT) cells are displayed (*n* =10–19/group, from three experiments). (**c,d**) Blood hemoglobin and platelet counts were assessed in animals (with ≥30% gene-corrected chimerism) on day 15 after infection (*n* = 7–9/group, except PGK.PRFmut where *n* = 2 due to early deaths, assessed across two experiments).

Optimal assessment of functional gene correction for perforin deficiency requires *in vivo* testing in the setting of a viral challenge. Therefore, we transduced bone marrow LSK cells from prf^−/−^ mice with PGK.PRF, PGK.PRFmut, and PRF.PRF and reconstituted irradiated prf^−/−^ mice. WT murine LSK cells were transplanted as a positive control, expressing endogenous murine perforin. After waiting 16 weeks for optimal immune reconstitution, we challenged these animals with LCMV infection. After LCMV challenge, prf^−/−^ mice develop marked elevation of serum IFN-γ levels, compared to WT animals, which leads to severe cytopenias, prostration, and death in most animals within 3 weeks. As expected, we observed that prf^−/−^ mice reconstituted with marrow expressing mutant (nonfunctional) perforin displayed much higher IFN-γ levels than those reconstituted with WT marrow. In comparison to animals reconstituted with mutant perforin (PGK.PRFmut), we observed that animals with ≥30% chimerism of perforin gene-corrected cells (either PGK.PRF or PRF.PRF, ranging from 33 to 92%, with a median chimerism of 72 and 47%, respectively) showed partial restoration of antigen-specific CTL killing and displayed significantly lower serum IFN-γ levels (**Figure 6a**,**b**). In addition, mice reconstituted with functional perforin displayed a significantly decreased drop in hemoglobin and platelet levels in comparison to mice reconstituted with mutant perforin (**Figure 6c**,**d**), suggesting that perforin gene transfer can at least partially prevent the cytopenias seen in virally driven HLH. Consistent with previously published data,^6^ animals with less than 30% gene-corrected chimerism did not display a clear trend toward correction of IFN-γ or other indices (data not shown). Because these animals were a small portion of all recipients (less than 1/3 of transplanted animals), we were not able to define a threshold of benefit more precisely. This initial proof-of-principle study demonstrates the feasibility of significantly improving immune regulation via lentiviral delivery of the perforin gene to HSCs. Further longer term studies including formal survival experiments are required for definitive confirmation of this approach.

## Discussion

FHL-2 is a devastating inherited disorder of childhood caused by mutations in the perforin gene. Current treatment options for this disease are limited, and permanent correction is currently only possible with hematopoietic stem cell transplantation. Hematopoietic stem cell gene therapy offers an alternative approach, and the present study shows that correction of the gene defect by lentiviral gene transfer into autologous HSCs leads to rescue of peripheral CD8^+^ T-cell cytotoxicity, partial rescue of NK cell cytotoxicity, and reversal of dysregulated IFN-γ secretion by CD8 T lymphoblasts *in vitro*.

Two lentiviral constructs containing either the PGK promoter or an endogenous perforin promoter, PRFprom, were tested in this study. Our expression data in RBL cells, which are able to process perforin, suggests that following gene transfer from these vectors, perforin is expressed, modified, and located in a similar way to perforin in WT cells.

The use of the PRFprom was designed to restrict perforin expression to mature T and NK cells and inhibit expression in stem cells where ectopic perforin expression may have unwanted side effects. The pattern of expression in a number of different cell lineages demonstrates the specificity of this promoter. While the PGK promoter was able to promote expression in all cell lines, the PRFprom was only active in the Jurkat T cell line and in the YT NK cell line (**Supplementary Figure S1**). The complete human perforin promoter region consists of 5.1 Kb and is composed of multiple sets of positive and neutral elements.^18^ For the construction of this vector, we used only a small portion of the endogenous promoter that included two repression sites (present −350 and −650 bp upstream the perforin gene) and the basal core promoter (present −244 bp upstream the perforin gene). The perforin promoter sequence used here does not contain *cis*-acting sequences that specifically enhance killer-lymphocyte transcription^21^ and are present in the full endogenous promoter sequence. Thus, further improvement of expression with this promoter is still possible with the addition of these sequences that enhance transcription.

The use of a specific lineage restricted promoter may offer safety benefits by avoiding perforin expression in stem cell and progenitor lineages. However, despite these concerns, in our studies, when perforin was expressed under the control of the ubiquitous PGK promoter in LSK cells, we did not observe any obvious adverse consequences in terms of progenitor colony formation or lineage commitment and development when compared to use of the PRF promoter that shows inhibition of expression in stem cells (**Figure 3c** and **Supplementary Figure S3**). Furthermore, the expression of perforin following PGK promoter progenitor cell transduction was still higher in activated T and NK cells in comparison to other peripheral cell types, as observed by the overall level of perforin expression in all tissues (**Supplementary Figure S4**). In the WT situation, transcriptional control and restriction of expression is provided mainly by a specific promoter^18,21,22^; however, other posttranscriptional mechanisms^23,24^ and posttranslational modifications also play a part in perforin expression.^25^ These studies confirm that there is a requirement for posttranscriptional/translational modification that is found only in T and NK cell lineages favoring the expression in these lineages even when the PGK promoter is used.

The PGK promoter has now been used in the context of self-inactivating LV vectors in a clinical trial of HSC gene therapy for metachromatic leukodystrophy with considerable efficacy^26^ and may have advantages for other hematopoietic diseases. If further studies suggest that high levels of stem cell expression of perforin is undesirable, then approaches such as the use of miRNA target sites that de-target perforin expression in stem cells could be used in conjunction with the PGK promoter in LV vectors.^27,28^

The fundamental defect in perforin deficiency is the failure of effector cell cytotoxicity that ultimately leads to hypercytokinemia and macrophage activation. In this study, we were able to show that effector cell correction following either peripheral or progenitor cell transduction led to recovery of cytotoxic function in both CD8^+^ T and NK cells (**Figures 2** and **4**). Interestingly, in CD8^+^ T cells, the level of recovery was complete and comparable to WT, while only partial correction was seen in NK cells despite equivalent levels of gene transfer in both cell lineages. Utilizing P14 TCR transgenic prf^−/−^ mice, we also demonstrated reconstitution of CD8^+^ T cell cytotoxicity against antigen-specific targets (**Figure 5**). The ability of restored perforin cytotoxicity to correct dysregulated IFN-γ secretion was demonstrated by the decreased IFN-γ production seen in PRF expressing CD8^+^ lymphoblasts when stimulated with anti-CD3 bound P815 targets (**Figure 4c**).

The reasons for the discrepancy in restoration of CD8 and NK cell killing are not clear, but previous studies have shown the importance of the CD8 population in controlling HLH. Indeed, murine and human transplant studies suggest that even low levels (~20%) of WT donor T-cell chimerism is sufficient to prevent HLH relapse^29^ and in the murine model protects against LCMV induced HLH.^6^ Thus, the engraftment of relatively low levels of functional gene corrected T cells could lead to significant clinical benefit. The extrapolation of this model to the clinical setting would be transduction of autologous CD34^+^ cells which are then reinfused into a conditioned host. The major advantage of this approach over allogeneic hematopoietic stem cell transplantation is the lack of graft-versus-host disease which is a significant complication in both matched and mismatched transplants. In addition, the safety of an autologous gene therapy procedure albeit with cytoreductive chemotherapy has been illustrated by the excellent 1 year survival in a number of gene therapy studies for Wiskott–Aldrich syndrome, chronic granulomatous disease, adrenoleukodystophy, and metachromatic leukodystrophy.^16,26,30,31^

Previous studies have shown how HLH may develop in prf^−/−^ mice after infection with LCMV.^5^ The features of HLH in the murine system accurately recapitulate human disease and include significant immune dysregulation with hypercytokinemia, macrophage activation, and cytopenias. In a viral infection such as LCMV, the CD8^+^ T cell cytotoxic and IFN-γ responses are vigorous, and the potential for immune-mediated pathology is substantial. We have challenged reconstituted mice with LCMV to ascertain if this gene correction approach will allow protection *in vivo*. In these experiments, we demonstrate that antigen-specific restoration of CTL killing is partially restored and hypersecretion of IFN-γ and the severity of cytopenias were significantly reduced, compared to untreated prf^−/−^ mice. The correction seen was partial but nevertheless significant. This level of correction was only seen in mice that had >30% gene-corrected cells. Together, these data suggest that full correction may require either higher number of engrafted, gene-modified cells and/or maximal perforin expression from gene corrected cells. Further studies are ongoing to address these issues, but these initial results provide encouraging data to support this approach.

Overall, this study demonstrates for the first time the potential for HSC gene therapy to correct an immune dysregulatory condition and rescue the functional defects associated with perforin deficiency. This can be achieved to a significant degree with the use of either an endogenous PRF promoter or a ubiquitous promoter such as PGK. The concept of using HSC gene transfer to correct immune developmental or function defects has been verified in other models^32^ but has not been tested for immune dysregulatory conditions. Perforin defects compose 30–40% of all genetic causes of FHL. We are aware of other projects that are developing gene therapy for defects in munc 13–4 which is the other major genetic cause of FHL (Cavazzana, personal communication), and thus, it is possible that in time a gene therapy option may be available for the majority of patients affected by FHL.

## Materials and Methods

***Vector construction.*** The steps involved in vector construction are described in **Supplementary Materials and Methods**.

***Mice.*** Perforin-deficient mice (prf^−/−^) were obtained from the Jackson Laboratory (Bar Harbor, ME) strain C57BL/6-Prf1tm1Sdz/J. All experiment procedures were approved by the Institutional Research Ethics Committee (Institute of Child Health, University College London, UK) and performed according to UK Home Office Animal Welfare Legislation. Murine studies at Cincinnati Children's Hospital Medical Center were conducted in accordance with Cincinnati Children's Hospital Medical Center approved protocol.

***Primary cells.*** Mouse primary NK cells were isolated from spleens of prf^−/−^ mice using CD49b (DX5) microbeads (Miltenyi Biotec, Woking, UK) following the manufacturer instructions. The cells were cultured in RPMI medium with 10% fetal calf serum, 1% Pen/Strep, 1 mmol/l 2-mercaptoethanol (Invitrogen), 1 mmol/l sodium pyruvate (Invitrogen), and 1000 U/ml IL-2 (Peprotech, Rocky Hill, NJ). Mouse primary CD8^+^ T cells were isolated from spleens of prf^−/−^ mice using CD8a (Ly-2) microbeads (Miltenyi Biotec) following the manufacturer's instructions. The cells were cultured in RPMI medium with 10% fetal calf serum, 1% Pen/Strep, 1 mmol/l 2-mercaptoethanol, 1 mmol/l sodium pyruvate, 30 U/ml IL-2, and 1 µg/ml anti-CD3 antibody (BD Biosciences, Oxford, UK). Both NK and CD8^+^ T cells were transduced by two rounds of spinoculation with vectors at an MOI of 50 at 1,800 g for 45 minutes at room temperature. After 72 hours, GFP expression was analyzed by flow cytometry, and cells were processed for cytotoxicity assays. The results presented show one representative experiment from a series of three experiments.

***Transduction and transplantation of LSK cells.*** Bone marrow cells were harvested from the femurs and tibias of prf^−/−^ mice, and the lineage negative (lin-ve) progenitors were isolated using a lineage cell depletion kit (Miltenyi Biotec). Lin-ve cells were blocked with 2.4 G antibody (BD Bioscience) for 15 minutes and then stained with streptavidin-fluorescein isothiocyanate, PE Ly-6A/E (Sca-1), and APC CD117 (c-kit; all from BD Biosciences) and sorted for the lin^-^sca-1^+^c-kit^+^ population (LSK cells) and the lin^-^sca-1^-^c-kit^-^ population (L^-^S^-^K^-^ cells) in a MoFlo XDP sorter (Beckman Coulter, Miami, FL). The LSK population was prestimulated overnight in StemSpan SFEM serum-free medium (StemCell Technologies, Grenoble, France) supplemented with 1% fetal bovine serum, 1% penicillin streptomycin, 100 ng/ml mSCF, 100 ng/ml mFLT-3L, and 20 ng/ml hTPO (all from Peprotech) and transduced at a MOI of 50 with the perforin vectors twice within a 12-hour interval. Transduced cells were harvested on the next day, and 2 × 10^4^ transduced LSK cells together with 4 × 10^4^ L^-^S^-^K^-^ irradiated cells (cultured in the transduction medium in parallel) were injected intravenously into the tail veins of lethally irradiated (6 Gy first day and 4 Gy second day) prf^-/-^ mice. Transduced LSK cells (2 × 10^4^) were left in culture, and 72 hours after transduction, GFP expression was analyzed by flow cytometry, and 1 week after transduction, cells were treated with benzonase and used to determine vector copy number. After 12 weeks, mice were sacrificed, and blood, bone marrow, thymus and spleen were removed for each mouse for analysis. From the spleens, NK cells and CD8^+^T cells were isolated and cultured as described in the Primary Cells section. NK cells and CD8^+^T cells were maintained in culture for 5 and 3 days, respectively and used for perforin intracellular staining and cytotoxicity assays. CD8^+^T cells cultured for 3 days were also used in an IFN-γ assay.

The reconstitution results presented in **Figure 4** show one representative experiment (experiment 2) from a series of three experiments where three mice were corrected with PGK.PRF and three mice were corrected with PRF.PRF transduced cells, the results from experiments 1 and 3 are shown as **Supplementary Figures S5 and S6**. A similar protocol was followed for the P14.prf^−/−^ LSK reconstitutions.

***^51^Cr release cytotoxicity assay and IFN-γ assay.*** For NK cell cytotoxicity, NK cells and ^51^Cr labeled RMA-S target cells (5 × 10^3^) were mixed in 96-well round bottom plates at various effector/target ratios and incubated for 4 hours at 37 °C. For CD8^+^ T-cell redirected killing assay, CD8^+^ T cells conjugated with anti-CD3 and ^51^Cr labeled P815 target cells were mixed in 96-well round bottom plates at various effector/target ratios and incubated for 4 hours at 37 °C. For antigen-specific CTL function, P14.prf^−/−^ splenocytes were stimulated *in vitro* with GP33 antigen for 48 hours and expanded 3 more days in IL-2 to generate CTL. The CTL were co-incubated with EL4 target cells labeled with GP33 and ^51^Cr in 96-well V bottom plates at various effector/target ratios and incubated 4 hours at 37 °C. For antigen-specific CTL function, CTLs were isolated from the spleens of mice 8 days after LCMV challenge. The CTLs were co-incubated with EL4 target cells labeled with GP33 and ^51^Cr in 96-well V-bottom plates at various effector/target ratios and incubated 4 hours at 37 °C. The release of ^51^Cr in the supernatant was measured with a γ-counter. All assays were done in triplicate.

For IFN-γ assay, 1 × 10^5^ CD8^+^ lymphoblasts labeled with 1 μg/ml anti-CD3, and 5 × 10^3^ P815 target cells were mixed in 96-well round bottom plates and incubated for 4 hours at 37 °C. The concentration of IFN-γ was determined by using enzyme linked immunosorbent assay kit obtained from R&D Systems (Minneapolis, MN) per the manufacturer's instructions.

**In vivo *LCMV challenge.*** Prf^−/−^ mice were injected with lentivirally transduced LSKs (2.5–5.0 × 10^4^ cells/animal) after lethal irradiation. After 16 weeks, animals were infected with LCMV-WE (200 pfu, i.p.). Animals were bled to assess serum IFN-γ levels at day 8 after infection and to perform complete blood counts on day 15 after infection.

***Statistics.*** Statistical significance was determined by using unpaired, two-tailed Student's *t*-tests.

**SUPPLEMENTARY MATERIAL** **Figure S1.** GFP expression in different cell lines after transduction 92 with PGK.PRF and PRF.PRF. **Figure S2.** Perforin expression and function in RBL-1 cells. **Figure S3.** Lineage development in reconstituted mice is not 107 affected by progenitor cell gene transfer. **Figure S4.** Transgene expression in different organs following 113 progenitor cell gene transfer. **Figure S5.** Lentiviral vector mediated HSC perforin gene transfer 117 restores T and NK cell cytotoxic function and reduces IFN-γ secretion by T 118 lymphoblasts *in vitro*- experiment 1. **Figure S6.** Lentiviral vector mediated HSC perforin gene transfer 131 restores T and NK cell cytotoxic function and reduces IFN-γ secretion by T 132 lymphoblasts *in vitro*- experiment 3. **Materials and Methods**
