# Supplementary material for: Perforin Gene Transfer Into Hematopoietic Stem Cells Improves Immune Dysregulation in Murine Models of Perforin Deficiency
Source: Mol Ther. 2015 Jan 20;23(4):737–45. doi: 10.1038/mt.2014.242 (PMC4395774; doi:10.1038/mt.2014.242)

Supplementary Material

Supplementary Materials and Methods

Vector construction: SIN lentiviral vectors (LV) incorporating the perforin cDNA were constructed using the HIV-1 vector backbone and a modified woodchuck post translational regulatory element (WPRE) as described previously[1]. The native human perforin gene was obtained by PCR amplification of cDNA using the following primers: 5' perforin Age: GCACCGGTGCCACCATGGCAGCCCGTCTGCTCCTCCT and 3' perforin Xho: TGCTCGAGTCACCACACGGCCCCACTCCGGTT with restriction sites underlined and then cloned into a pRRL backbone containing the human phosphoglycerate kinase promoter (PGK). The perforin gene and the PGK promoter were cloned from the pRRL.PGK.PRF into a pHR'SINcPPT.SEW.SFFV.IRES.GFP backbone containing the internal ribosomal entry site (IRES) and GFP after the removal of the SFFV promoter sequence to create pPGK.PRF.I.GFP. The human perforin promoter was obtained by PCR amplification of a ~1.6Kb human DNA region using the following primers: PRF Nhe 5'-CAGCTAGCGAATTCCAAAGTCCTCTCTTTGATTTTAT-3' and PRF Age 5'-GCACCGGTGGCATCAGCCCCCCAGGCAGCCCACT-3' with restriction sites underlined, and then cloned into a pRRL backbone from which it was removed together with the perforin cDNA into a pHR'SINcPPT.SEW.IRES.GFP backbone to create pPRF.PRF.I.GFP. A vector containing a non-functional mutated version of the perforin gene (PRFmut) was also constructed. This was done by cloning a perforin sequence with a missense mutation (PRF-T435M)[2] under the control of the PGK promoter to create pPGK.PRFmut.I.GFP.

Production of lentiviral particles and titration: Lentiviral supernatants were produced by transient co transfection of vector plasmid, envelope plasmid and packaging plasmids into 293T cells in the presence of polyethylamine (Sigma Aldrich, Dorset, UK). The VSV-G envelope plasmid (pMD.G2) and second-generation packaging

plasmid (pCMVΔ8.91) were produced by Plasmid Factory (Bielfield, Germany). The viral supernatants were concentrated by ultracentrifugation at 98,000g for 2hr at 4°C. The viral titer was determined by transducing RBL-1 cells with serial dilutions of the viruses. The expression was monitored by flow cytometry detecting GFP.

Cell lines: RBL-1 and 293T cell lines were maintained in Dulbecco's modified Eagle's medium (Invitrogen, Paisley, UK). RMA-S, P815, Jurkat, U937, K562, LCL cell lines were cultured in RPMI 1640 medium (Roswell Park Memorial Institute medium, Invitrogen). All media were supplemented with 10% fetal bovine serum (FBS) and 100U/ml penicillin/streptomycin (Invitrogen).

Western blot analysis and confocal staining: Cell lysates from transduced RBL cells were resolved on a 10% sodium dodecyl sulfate–polyacrylamide gel electrophoresis (SDSPAGE;Tris-Glycine) gel under non reducing conditions, which was then analyzed for perforin or actin expression by immunoblotting with anti-perforin antibody 2d4 (kindly provided by Dr. Griffiths) or anti-actin antibody (Sigma), followed by the secondary horseradish peroxidase–linked anti–mouse or anti–rabbit immunoglobulin (Sigma). The signal was detected by chemiluminescence (Amersham Biosciences, Little Chalfont, UK).

Transduced RBL-1 cells were plated on glass coverslips coated with poly-L-lysine (Sigma) and incubated 1h at 37°C. Cells were fixed by incubation in 4% paraformaldehyde for 10min. The cells were incubated with anti perforin 1 (H-315, Santa Cruz) in permeabilizing buffer (PBS with 0.01% triton) for 1h and with secondary antibody PE-Cy7 anti rabbit (Sigma) for 30min, washed and mounted on slides with antifading medium. Samples were observed with a Zeiss LSM710 inverted confocal microscope.

Real-time qPCR: Genomic DNA was isolated from cells using the DNAeasy extraction kit (Qiagen, West Sussex, UK). Average vector copy number per cell was determined by WPRE quantitative PCR in Platinum Quantitative PCR Supermix-UDG with Rox

(Invitrogen). The titin gene was used as an endogenous gene control. The primer and probe sequences for mutant WPRE were as follows: forward, 5'-TGGATTCTGCGCGGGA-3'; and reverse, 5'-GAAGGAAGGTCCGCTGGATT-3'; probe, 5'-FAM_CTTCTGCTACGTCCCTTCGGCCCT_TAMRA-3'. The primer and probe sequences for titin were as follows: forward, 5'-AAAACGAGCAGTGACCTGAGG-3'; and reverse, 5'-TTCAGTCATGCTGCTAGCGC-3'; probe, 5'-FAM_TGCACGGAATCTCGTCTCAGTC_TAMRA-3'.

Immunophenotyping by flow cytometry: Flow cytometric analyses were performed on cells obtained from blood, bone marrow, thymus and spleen. Blood was lysed and leukocytes were washed with PBS. Cells were incubated for 30 minutes at room temperature in PBS containing 0.5% BSA and antibodies against CD4, CD8, NK1.1 or B220 directly conjugated with APC (all BD Biosciences). Subsequently cells were washed and measured on a CyAn™ ADP Flow Cytometer (Dako, Carpinteria, CA). Spleen and thymus cells were evaluated similarly. Additionally GFP expression was measured in mice treated with the PGK.GFP, PGK.PRF, PGK.PRFmut and PRF.PRF.

Determination of perforin expression: For intracellular perforin staining, 1×10^6 cells were fixed with 1% PFA in PBS for 20 min at room temperature and incubated 10 min with BD perm/wash buffer 1X (BD Biosciences). Cells were washed 2 times with BD perm/wash buffer 1X and then stained with PE-conjugated mouse anti-human perforin mAb (δG9, IgG2b; BD Biosciences) in BD perm/wash buffer 1X for 30 min at room temperature. Unbound antibodies were removed by washing with perm/wash buffer 1X and fixed again with 1% PFA. At least 25,000 cells were analyzed for each sample by flow cytometry.

Supplementary references

1. Demaison C, Parsley K, Brouns G, Scherr M, Battmer K, Kinnon C, et al. High-level transduction and gene expression in hematopoietic repopulating cells using a human immunodeficiency [correction of imunodeficiency] virus type 1-based lentiviral vector containing an internal spleen focus forming virus promoter. Hum Gene Ther 2002;13(7):803-13.

2. Urrea Moreno R, Gil J, Rodriguez-Sainz C, Cela E, LaFay V, Oloizia B, et al. Functional assessment of perforin C2 domain mutations illustrates the critical role for calcium-dependent lipid binding in perforin cytotoxic function. Blood 2009;113(2):338-46.

Supplementary Figure legends

Supplementary Figure S1: GFP expression in different cell lines after transduction with PGK.PRF and PRF.PRF. The different cell lines were transduced with the vectors at an MOI of 50 and GFP and PRF expression were observed by FACS after 5 days. The values observed are normalized to the copy number. U937 is a monocytic cell line, LCL is a B lymphocytic cell line, 293T is an embryonic kidney cell line, K562 is a myeloid cell line, Jurkat is a T cell line and YT is a NK cell line.

Supplementary Figure S2: Perforin expression and function in RBL-1 cells (a) Detection of perforin in YT cells as well as in RBL-1 cells transduced with PGK.PRF by western blotting following non-reducing SDS-PAGE. All forms of perforin were visualized in the 2 cell lines by anti-prf 2d4 antibody. The negative control is non-transduced RBL-1 cells. (b) Visualization of perforin by confocal microscopy in RBL-1 cells transduced with PGK.PRF and in normal YT cells. Perforin staining is shown in white.

Supplementary Figure S3: Lineage development in reconstituted mice is not affected by progenitor cell gene transfer. Percentage of T cells, NK cells, B cells and GR1 cells in the spleen, thymus, and blood of $prf^{-/-}$ mice reconstituted with LSK cells transduced with PGK.GFP, PGK.PRF and PRF.PRF, $prf^{-/-}$ mice non reconstituted and WT mice.

Supplementary Figure S4: Transgene expression in different organs following progenitor cell gene transfer. GFP and perforin expression in bone marrow, thymus, blood, spleen and spleen derived and stimulated $CD8^{+}$ T cells and NK cells from prf-/- mice reconstituted with LSK cells transduced with (a) PGK.PRF and (b) PRF.PRF.

Supplementary Figure S5: Lentiviral vector mediated HSC perforin gene transfer restores T and NK cell cytotoxic function and reduces IFN-γ secretion by T lymphoblasts *in vitro*- experiment 1. (a) ^{51}Cr release from RMA-S cells co-incubated with NK cells from mice reconstituted with LSK cells transduced with PGK.GFP, PGK.PRF and PRF.PRF, prf$^{-/-}$ and WT mice. (b) ^{51}Cr release from anti-CD3 bound P815 cells co-incubated with CD8^{+} T cells from mice reconstituted with LSK cells transduced with PGK.GFP, PGK.PRF and PRF.PRF, prf$^{-/-}$ and WT mice. The p values correspond to both the comparisons between the PGK.PRF and the PRF.PRF groups with the PGK.GFP group. The highest p value is shown for each point. (c) IFN-γ production by CD8^{+} lymphoblasts derived from prf$^{-/-}$ mice reconstituted with LSK cells transduced with PGK.GFP, PGK.PRF and PRF.PRF, from prf$^{-/-}$ and from WT mice after co-incubation with anti-CD3 bound P815 cells for 4 hours. For the 3 assays n=3 for each group and the error bars represent the SD.

Supplementary Figure S6: Lentiviral vector mediated HSC perforin gene transfer restores T and NK cell cytotoxic function and reduces IFN-γ secretion by T lymphoblasts *in vitro*- experiment 3. (a) ^{51}Cr release from RMA-S cells co-incubated with NK cells from mice reconstituted with LSK cells transduced with PGK.GFP, PGK.PRF and PRF.PRF, prf$^{-/-}$ and WT mice. (b) ^{51}Cr release from anti-CD3 bound P815 cells co-incubated with CD8^{+} T cells from mice reconstituted with LSK cells transduced with PGK.GFP, PGK.PRF and PRF.PRF, prf$^{-/-}$ and WT mice. The p values correspond to both the comparisons between the PGK.PRF and the PRF.PRF groups with the PGK.GFP group. The highest p value is shown for each point. (c) IFN-γ production by CD8^{+} lymphoblasts derived from prf$^{-/-}$ mice reconstituted with LSK cells transduced with PGK.GFP, PGK.PRF and PRF.PRF, from prf$^{-/-}$ and from WT mice after co-incubation with anti-CD3 bound P815 cells for 4 hours. For the 3 assays n=3 for each group and the error bars represent the SD.

Supplementary Figure S1

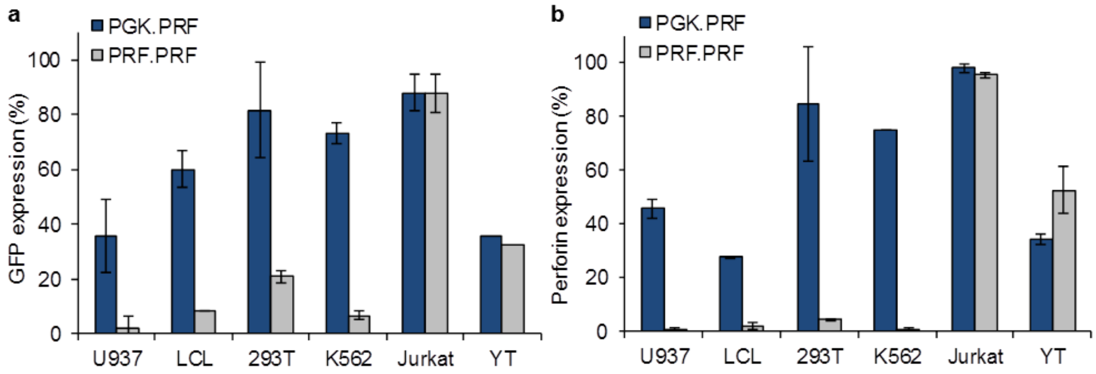

Supplementary Figure S2

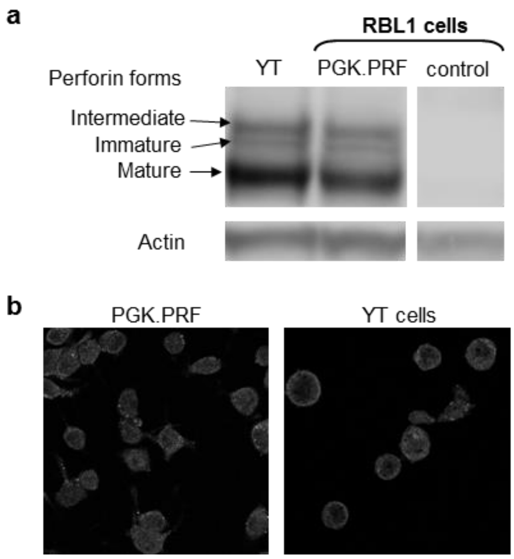

Supplementary Figure S3

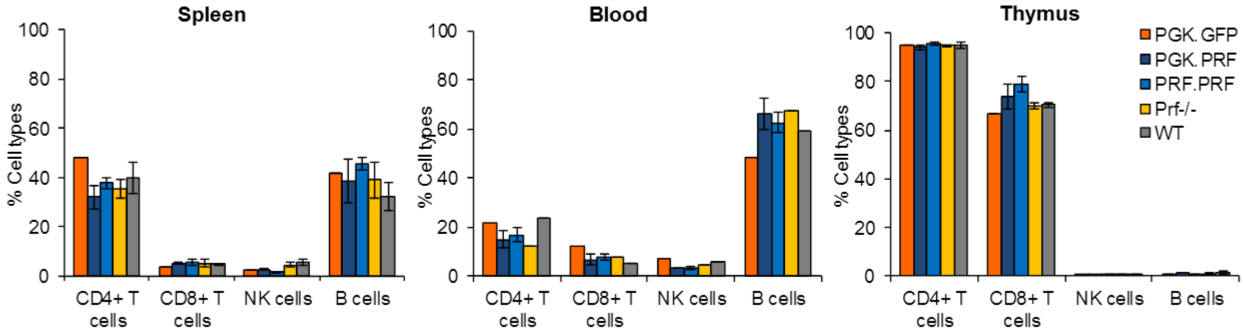

Supplementary Figure S4

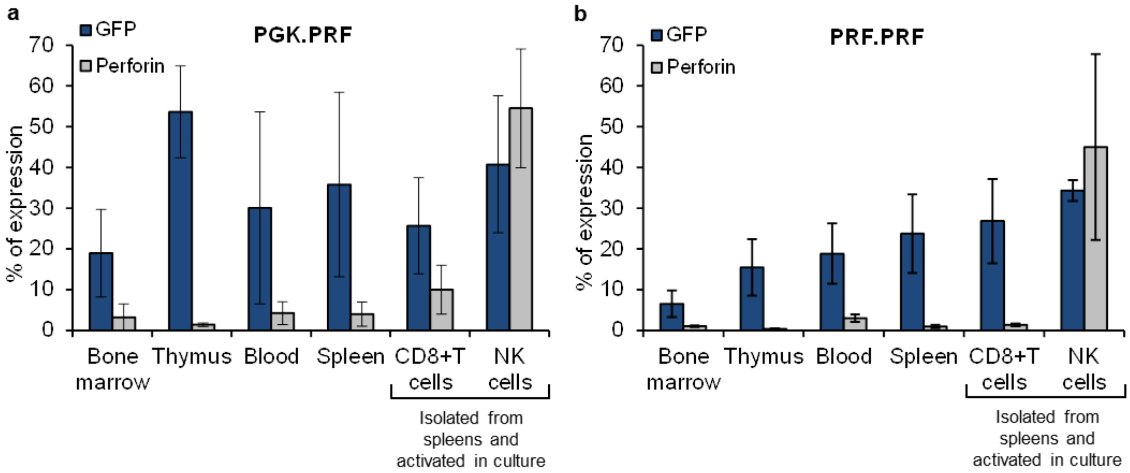

Supplementary Figure S5

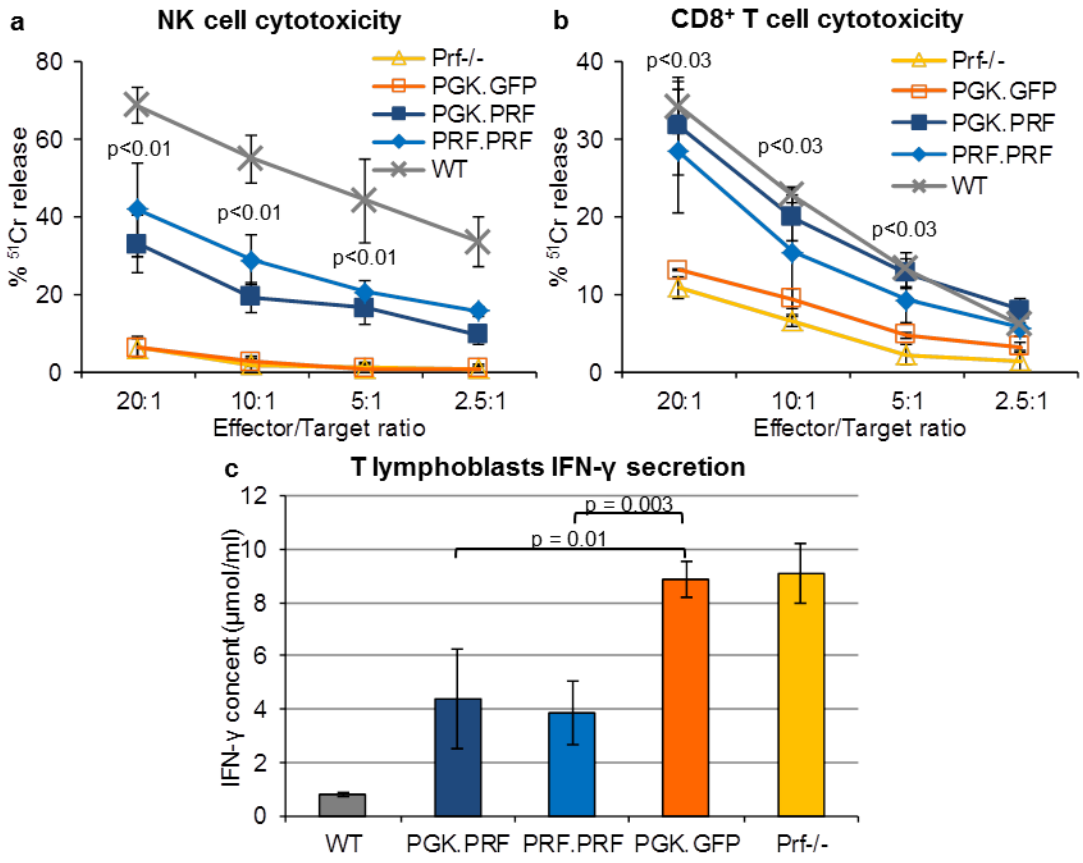

Supplementary Figure S6

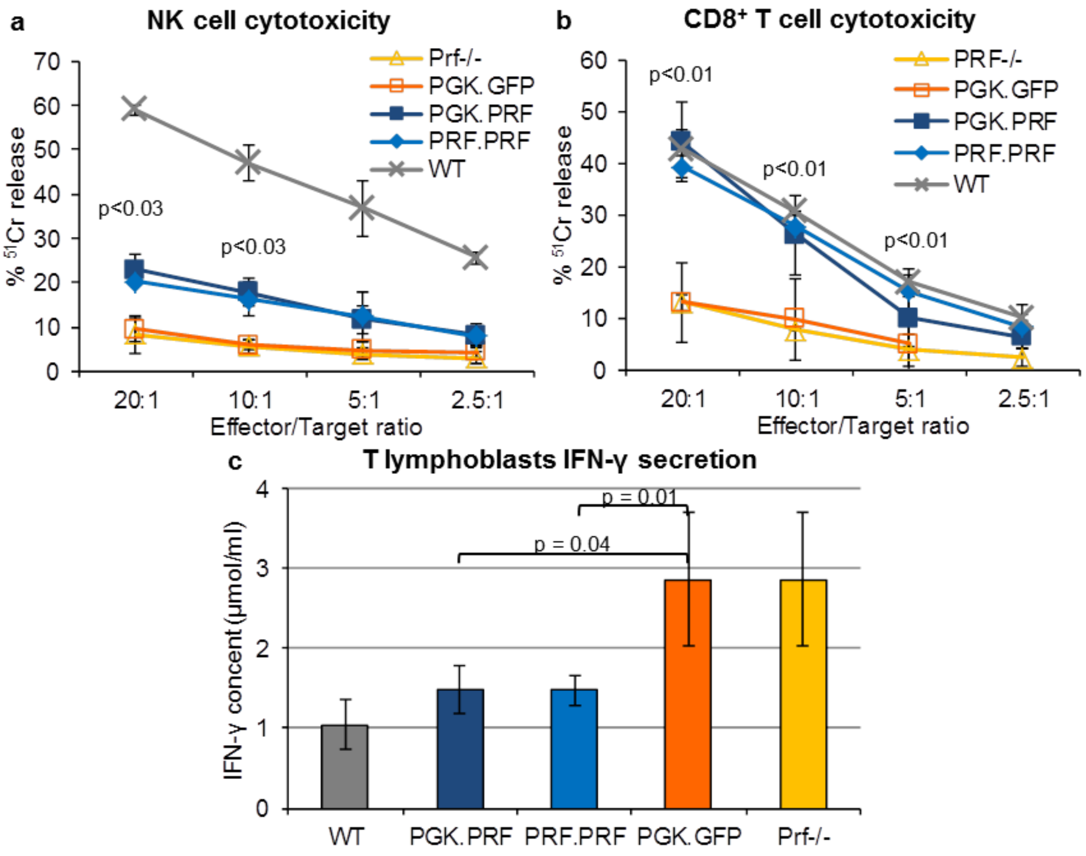

Supplement: Supplementary Information [file mt2014242x1.pdf]
